# Relationships between glucose variability with white matter hyperintensity and cerebrovascular abnormalities

**DOI:** 10.20945/2359-4292-2026-0062

**Published:** 2026-06-08

**Authors:** Cheng-Chieh Lin, Chia-Ing Li, Chiu-Shong Liu, Chih-Hsueh Lin, Shing-Yu Yang, Tsai-Chung Li

**Affiliations:** 1 School of Medicine, College of Medicine, China Medical University, Taichung, Taiwan; 2 Department of Medical Research, China Medical University Hospital, Taichung, Taiwan; 3 Department of Family Medicine, China Medical University Hospital, Taichung, Taiwan; 4 Department of Public Health, College of Public Health, China Medical University, Taichung, Taiwan; 5 Department of Audiology and Speech-Language Pathology, Asia University, Taichung, Taiwan

**Keywords:** Type 2 diabetes, glycemic variability, white matter hyperintensity, cerebrovascular abnormalities

## Abstract

**Objective:**

Epidemiological studies have revealed that glucose variability (GV) is a predictor of stroke, cognitive impairment, and dementia in patients with type 2 diabetes mellitus (T2DM). However, evidence on the associations of GV with white matter hyperintensity (WMH) and cerebrovascular abnormalities remains scarce. This study aimed to explore the relationships of GV with WMH and cerebrovascular abnormalities using epidemiological and Mendelian randomization (MR) approaches. The MR approach was used to assess the effects of genetic proxies for GV on MRI outcomes.

**Subjects and methods:**

This cross-sectional study was conducted at a medical center where patients with T2DM were recruited. The measures for fasting plasma glucose (FPG) and HbA1c variability included the standard deviation, coefficient of variation, average real variability (ARV), and variability independent of the mean (VIM). Brain magnetic resonance images were analyzed to assess WMHs and cerebrovascular abnormalities. For MR, instrumental variables were used to assess the causal relationships between glycemic variability and outcome based on two-stage regression analysis.

**Results:**

This study included 2,247 subjects, of whom 1,122 had WMH and 957 had cerebrovascular abnormalities. We found 80 independent single-nucleotide polymorphisms associated with GV but not with WMH or cerebrovascular abnormalities, which were subsequently used as genetic instruments. Genetically increased, unweighted FPG-VIM was linked with WMH (odds ratio 1.17 [95% CI 1.08, 1.27] per standard deviation). All genetically increased, unweighted and weighted GV measures were associated with cerebrovascular abnormalities, except FPG-ARV.

**Conclusion:**

Our study provided evidence that genetically predicted GV was associated with WMH and cerebrovascular abnormalities, supporting a potential causal link under MR assumptions.

## INTRODUCTION

A global pandemic of type 2 diabetes is currently underway, driven by factors such as rapid urbanization, an aging global population, and rising obesity rates associated with energy-dense diets and sedentary lifestyles. This public health crisis affects low-, middle-, and high-income countries. The global prevalence of type 2 diabetes increased markedly from 151 million in 2000 to 537 million by 2021, significantly exceeding the earlier 1998 projection of 300 million by 2021 (^1^,^2^). Type 2 diabetes is linked to a range of complications, including macrovascular conditions - such as coronary artery disease, stroke, and peripheral arterial disease - and microvascular complications. These outcomes, largely attributed to atherosclerotic processes, impose a considerable burden on health care systems worldwide (^3^-^5^). Cardiovascular diseases, including heart failure, coronary heart disease, cerebrovascular disease, and other cardiac conditions, remained the leading cause of mortality worldwide and accounted for more than 30% of all deaths in 2016 (^6^). A comprehensive meta-analysis of 102 prospective studies revealed that compared with nondiabetic individuals, individuals with diabetes face a 2- to 4-fold increased risk of ischemic stroke and coronary heart disease and a 1.5- to 3.6-fold increase in all-cause mortality. These risks are further exacerbated by poor glycemic control (^7^).

Glycemic control has long been considered a fundamental strategy for mitigating diabetic complications, with glycosylated hemoglobin (HbA1c) widely recognized as the “gold standard” biomarker for long-term glycemic regulation. However, randomized controlled trials have indicated that reduced blood glucose levels do not necessarily correspond to a decreased incidence of diabetic complications (^8^-^10^). One plausible explanation is that these studies did not adequately account for glucose variability-marked fluctuations in blood glucose levels over time-which may play a critical role in the development of diabetic complications. The concept of “glucose variability” or “glycemic variability” has garnered increasing attention from the research community. Mounting evidence suggests that visit-to-visit glucose fluctuations are significantly associated with diabetes-related complications and mortality. To date, experimental evidence confirming the harmful effects of glucose variability is lacking. Thus, further investigations using methodologies capable of establishing experimental causality are warranted to clarify the role of glucose variability in the pathogenesis of diabetes-related complications. Mendelian randomization (MR) provides an alternative methodological framework for inferring causal relationships and generating evidence analogous to experimental results.

Quantitative and noninvasive in nature, brain magnetic resonance imaging (MRI) has become increasingly accessible in clinical settings and is frequently utilized as a surrogate endpoint for identifying novel determinants of brain health (^11^). One commonly examined MRI parameter is white matter hyperintensity (WMH) volume, which represents lesions in the white matter of the brain. These lesions make the white matter appear hyperintense on fluid-attenuated inversion recovery (FLAIR) sequences. Brain MRI, particularly WMH assessment, is widely regarded as a marker of cerebral small vessel disease (^12^). Increased WMH volumes have been associated with increased risks of Alzheimer’s disease, incident stroke, all-cause mortality, and cognitive decline (^13^). Advanced brain MRI techniques enable the visualization and quantification of structural and microvascular abnormalities, thereby facilitating the early detection of cerebrovascular disease even in asymptomatic individuals.

Previous studies that employed traditional epidemiologic or MR designs to examine the association of glucose level or variability with WMH and cerebrovascular abnormalities have been limited (^14^-^16^). One study explored the association between HbA1c levels and WMH in the general population (^16^). Research on glucose variability has focused primarily on individuals with type 1 diabetes (^14^), whereas another study investigated a small sample of older adults with type 2 diabetes who carried the APOE4 allele (^15^). One MR study investigated the association between HbA1c levels and WMH. Considering the limited number of studies and their small sample sizes, we aimed to assess the associations between visit-to-visit glucose variability and brain MRI measurements using observational epidemiologic and MR approaches. The MR approach was used to assess the effects of glucose variability (GV) on MRI outcomes.

## SUBJECTS AND METHODS

### Study subjects

**Figure 1:**
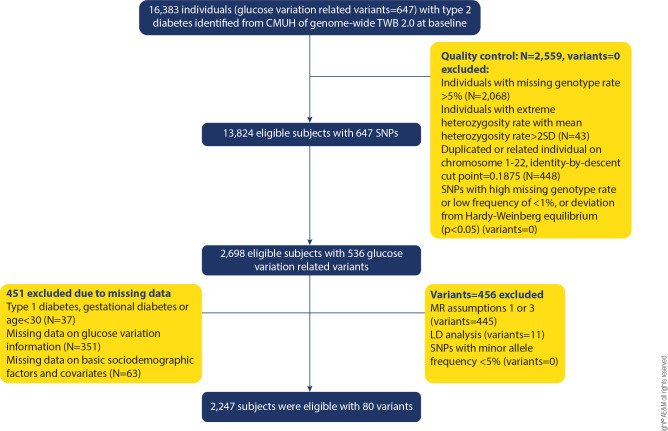
The study flowchart for study subject and SNP selection.

A retrospective cohort study was conducted among individuals with type 2 diabetes who were enrolled in the Diabetes Care Management Program (DCMP) at China Medical University Hospital (CMUH). The inclusion criterion was individuals diagnosed with diabetes, identified by the International Classification of Diseases, Ninth Revision, Clinical Modification (ICD-9-CM) code 250. The exclusion criteria included individuals younger than 30 years, those with type 1 diabetes (ICD-9-CM codes 250.x1/x3), and those diagnosed with gestational diabetes (ICD-9-CM code 648.83). Participants were recruited between November 2001 and June 2022. The index date was defined as the date of the first available brain MRI measurement. Individuals lacking data on brain MRI, laboratory tests, or dietary records were excluded from the analysis (**Figure 1**). Measurements for other study variables were obtained from the date closest to the index date. This study was approved by the Ethical Review Board of the CMUH (CMUH112-REC1-007).

### Data source

The data for this study were derived from the computerized databases of the Clinical Research Data Repository (CRDR) and the DCMP at CMUH in Taichung, Taiwan. Established in 2017, the CMUH-CRDR integrates data from multiple clinical sources with the goal of improving healthcare quality by consolidating trackable patient information generated during routine clinical practice. This repository includes comprehensive data, such as laboratory and physiological measurements, prescription records, hospitalizations, surgical procedures, and emergency department visits. It also contains whole-genome genotyping data generated using Affymetrix Axiom Genome-Wide TPM array chips (Affymetrix, Santa Clara, CA, USA). Moreover, the DCMP database contains information on individuals diagnosed with diabetes based on the diagnostic criteria set forth by the American Diabetes Association. Eligible patients were invited to enroll in the program by their physicians. Only healthcare professionals who have completed the required clinical education and training programs were authorized to recruit patients into the DCMP.

### Measurements

Upon enrollment in the DCMP, patients underwent a series of assessments, such as anthropometric measurements and blood and urine tests. Information on dietary habits, lifestyle factors, and past or current medical conditions was collected by trained case nurses using a standardized computerized questionnaire. Detailed definitions of all study variables are provided in the sections below.

#### Key predictor variables

In this study, the primary predictor variable was glucose variability, which was using the following metrics: variability independent of the mean (VIM), average real variability (ARV), coefficient of variation (CV), standard deviation (SD), and slope of HbA1c and fasting plasma glucose (FPG). These indices were calculated on the basis of annual outpatient measurements over the 1-year period preceding the index date. As recommended in prior research, variability estimates were adjusted using a weighting factor equal to the reciprocal of √(n/(n - 1)) to account for differences in the number of glucose measurements per individual (^17^). Glucose variability metrics were derived only for participants with at least two recorded measurements of HbA1c and FPG.

### Covariates

The demographic and family history variables included participants’ sex; age at entry into the DCMP. Lifestyle variables, as recorded in the DCMP dataset, included physical activity, alcohol consumption, smoking, passive smoking, and habitual dietary intake.

The medication variables included the use of oral hypoglycemic agents and insulin. Oral agents were categorized into seven classes as follows: meglitinides, sulfonylureas, biguanides, insulin sensitizers, dipeptidyl peptidase-4 inhibitors, α-glucosidase inhibitors, and other compounds. Insulin types included insulin aspart, regular insulin, neutral protamine hagedorn, levemir, glucagon-like peptide-1 analogs, and lantus. The use of other medications, including antihypertensives (e.g., calcium channel blockers), nephropathy medications, and lipid-lowering agents (e.g., statins), was recorded electronically and classified as “yes” or “no”, baseline comorbidities and diabetic complications were also classified dichotomously. Comorbidities included hyperlipidemia, hypertension, and obesity. Chronic complications included stroke, peripheral neuropathy, peripheral vascular disease, retinopathy, diabetic foot, nephropathy, and amputation. Acute complications included hyperglycemic hyperosmolar nonketotic coma (HHNK), severe hypoglycemia, and diabetic ketoacidosis.

### Instrumental variables

#### Single-nucleotide polymorphism (SNP) genotyping in MR analysis

The SNP data used as instrumental variables were obtained from the iHi Genomics, CMUH-CRDR (^18^) and were based on DNA samples genotyped using the TPM array and analyzed with the Axiom Genome-Wide Array Plate System (Affymetrix, Santa Clara, CA, USA). Quality control included assessment of Hardy-Weinberg equilibrium using PLINK v2.0 (^19^), and genotype imputation was performed with IMPUTE2 software (^20^) using the 1000 Genomes Project as the reference panel. Genetic variants were selected under the guidance of previous studies employing candidate gene and genome-wide association study (GWAS) approaches for HbA1c (^21^-^23^) and FPG (^24^,^25^). A curated list of SNPs associated with HbA1c and FPG was constructed. For HbA1c, the variants in genes involved in glycemic pathways (e.g., *CDKAL1, DGKB, GCK, SLC30A8, MTAP*, and *KL*) and nonglycemic pathways (e.g., *SPATS2L, TRAM2-AS1, RPA2P2, KCNKS*, and *ARAP3*) were included. For FPG, SNPs from loci such as *HFE, MYB, ANK1, HK1*, and *PHB2* were considered. The MR literature was further searched for SNPs (^16^,^26^-^32^), especially those linked to the potential biological mechanisms of glucose variability. A total of 536 SNPs were found in the GWAS data from iHi Genomics. The SNPs not identified in the iHi genomics dataset, with minor allele frequencies < 5%, violating MR assumptions 1 and 3 (SNP = 445), and in high linkage disequilibrium (SNP = 11) were removed. Finally, 80 SNPs were included in the analysis. Approval was obtained from the Human Research Committee of CMUH (CMUH112-REC1-007), and this study was conducted in accordance with relevant regulations and guidelines.

#### Assessment of brain MRI scans

Brain MRI scans were performed using a 3.0 Tesla scanner (SIGNA HDxt), and imaging data were retrieved from the hospital’s electronic medical records. Axial T2-weighted FLAIR sequences were used for the assessment of WMHs. WMHs were defined as those with subcortical or periventricular hyperintensities visible on FLAIR images and were assessed visually by a board-certified radiologist, who was blinded to the clinical information. WMH was operationalized as a binary variable, defined as “yes” if any WMH lesion was identified, and “no” otherwise. The interand intrarater reliability of cerebrovascular findings were evaluated on a sample of 60 MRI images, yielding kappa statistics exceeding 0.80 across all assessed conditions, indicating high agreement. Cerebrovascular abnormalities were also assessed by a radiologist. Cerebrovascular abnormalities included stenosis or occlusion of major intracranial or internal carotid arteries, aneurysms, lacunar or small infarctions, intracerebral hemorrhage, and lobar infarctions.

### Statistical analysis

Descriptive statistics are reported as the means and standard deviations for continuous variables and as frequencies and proportions for categorical variables. Bivariate analyses were conducted using chi-square tests for categorical variables and two-sample *t*-tests for continuous variables. Multiple logistic regression was used to adjust for age, sex, lifestyle behaviors (smoking, alcohol consumption, physical activity, and BMI), diabetes-related variables (duration of diabetes and type of hypoglycemic drug use), comorbidities (stroke, hypertension, obesity, coronary artery disease, hyperlipidemia, peripheral neuropathy, neuropathy and nephropathy), drug-related variables (cardiovascular medications, hyperlipidemia medications, and hypertension medications) and biomarkers (HDL-C, TG, LDL-C, TC, eGFR, FPG, and HbA1c). All statistical analyses were performed using SAS version 9.4 (SAS Institute, Cary, NC) with two-tailed *p*-values and a significance threshold of 0.05.

### MR analysis

Quality control excluded individuals with high genotyping missingness, extreme heterozygosity, or genetic relatedness. SNPs were removed for low minor allele frequency, high missingness, or Hardy-Weinberg disequilibrium (tested via chi-square in controls). MR assumptions were assessed as follows: For relevance (assumption 1), SNP associations with glucose variability were tested using ANOVA and linear regression (additive model); for exclusion restriction (assumption 3), chi-square tests ensured no association with brain MRI variables. Only SNPs meeting both criteria were used to construct weighted and unweighted genetic risk scores (GRSs).

Before the GRSs were constructed, linkage disequilibrium (LD) was assessed using pairwise r^2^ in Haploview v4.2; for SNP pairs with r^2^ > 0.8, one was retained on the basis of prior associations with glucose traits. The weighted GRS was calculated by summing the products of minor allele counts and their regression coefficients. GRS values were divided into quartiles to assess linearity, and analyzed as a continuous variable. Linear regression was used to test the association between GRSs and glucose variability (assumption 1), whereas multinomial logistic regression was used to assess the associations with covariates (assumption 2). Assumption 3 was tested via logistic regression with brain MRI variables.

MR analyses used two-stage instrumental variable regression with multivariable adjustment to estimate the causal effect of glucose variability on brain MRI outcomes. In stage one, glucose variability was predicted from the weighted GRS. In stage two, these predicted values were used in logistic regression with brain MRI measures. Models adjusted for first-stage residuals, covariates violating MR assumption 2, and the top 10 genetic principal components. Horizontal pleiotropy was assessed using MR-Egger regression, and between-instrument heterogeneity was evaluated using Cochran’s Q statistics under the inverse-variance weighted framework. All tests were two-sided with p < 0.05.

## RESULTS

### Baseline characteristics of the study subjects

A total of 2,247 individuals with type 2 diabetes from the Taichung Diabetes Study were included, with a mean baseline age of 65.00 years (SD = 11.14). Among these participants, 1,122 (49.9%) had WMHs and 957 (42.6%) had cerebrovascular abnormalities. **Table 1** shows the characteristics of the study subjects grouped by WMH volume and cerebrovascular abnormalities. Individuals with WMH were significantly older and had a low proportion of men and smokers. They also had a long mean duration of diabetes, a high prevalence of cardiovascular and antihypertensive medication use, and low mean LDL-C level and eGFR. Moreover, significantly more individuals with cerebrovascular abnormalities were older and had a high proportion of men and a greater prevalence of injection use, oral hypoglycemic drug use, hypertension, stroke, coronary artery disease, and neuropathy. They also had a long mean duration of diabetes, high HbA1c levels, a high prevalence of cardiovascular and hypertension medication use, and low mean HDL-C level and eGFR.

**Table 1:** Comparisons of sociodemographic factors, lifestyle behaviors, diabetes-related variables, glucose variation and comorbidities according to WMH or cerebrovascular abnormality

Variables	WMH, N (%)		P value		Cerebrovascular abnormality, N (%)		P value
	No (N=1,125)	Yes (N=1,122)			No (N=1,290)	Yes (N=957)	
Sociodemographic factors							
Age, years, mean±SD	62.10±11.29	67.90±10.68	<0.001		64.07±11.50	66.24±11.06	<0.001
Gender			0.02				0.02
Men	634 (56.36)	576 (51.34)			666 (51.63)	544 (56.84)	
Women	491 (43.64)	546 (48.66)			624 (48.37)	413 (43.16)	
Education level (years)			<0.001				0.01
0	163 (42.23)	223 (57.77)			209 (54.15)	177 (45.85)	
1-6	250 (42.09)	344 (57.91)			323 (54.38)	271 (45.62)	
7-12	426 (54.62)	354 (45.38)			449 (57.56)	331 (42.44)	
≥12	286 (58.73)	201 (41.27)			309 (63.45)	178 (36.55)	
APOE4 (rs429358)			0.03				0.28
TT	902 (80.18)	939 (83.69)			1071 (83.02)	770 (80.46)	
CT	219 (19.47)	175 (15.60)			213 (16.51)	181 (18.91)	
CC	4 (0.36)	8 (0.71)			6 (0.47)	6 (0.63)	
Lifestyle behaviors							
Smoking			0.03				0.72
No	963 (85.6)	996 (88.77)			1128 (87.44)	831 (86.83)	
Yes	162 (14.4)	126 (11.23)			162 (12.56)	126 (13.17)	
Alcohol drinking			0.40				0.49
No	1068 (94.93)	1055 (94.03)			1223 (94.81)	900 (94.04)	
Yes	57 (5.07)	67 (5.97)			67 (5.19)	57 (5.96)	
Physical activity			0.43				0.95
No	600 (53.33)	618 (55.08)			698 (54.11)	520 (54.34)	
Yes	525 (46.67)	504 (44.92)			592 (45.89)	437 (45.66)	
BMI, kg/m^2^, mean±SD	26.29±4.34	26.10±3.9	0.28		26.32±4.22	26.04±3.99	0.11
Diabetes-related variables							
Duration of diabetes, years, mean±SD	6.18±7.54	7.39±8.09	<0.001		6.38±7.48	7.33±8.28	0.005
Type of hypoglycemic drug use			0.59				0.02
No	62 (5.51)	48 (4.28)			73 (5.66)	37 (3.87)	
OAD	905 (80.44)	910 (81.11)			1049 (81.32)	766 (80.04)	
Inject insulin	16 (1.42)	16 (1.43)			21 (1.63)	11 (1.15)	
Both	142 (12.62)	148 (13.19)			147 (11.40)	143 (14.94)	
Comorbidity							
Stroke	75 (6.67)	87 (7.75)	0.36		41 (3.18)	121 (12.64)	<0.001
Hypertension	422 (37.51)	445 (39.66)	0.32		466 (36.12)	401 (41.9)	0.006
Obesity	248 (22.04)	243 (21.66)	0.86		289 (22.4)	202 (21.04)	0.49
Coronary artery disease	83 (7.38)	63 (5.61)	0.11		68 (5.27)	78 (8.15)	0.008
Hyperlipidemia	296 (26.31)	286 (25.49)	0.69		347 (26.9)	235 (24.56)	0.23
Peripheral neuropathy	75 (6.67)	83 (7.4)	0.55		92 (7.13)	66 (6.9)	0.89
Neuropathy	23 (2.04)	22 (1.96)	1.00		16 (1.24)	29 (3.03)	0.005
Nephropathy	54 (4.80)	73 (6.51)	0.10		66 (5.12)	61 (6.37)	0.24
Drug-related variables							
Cardiovascular medications	312 (27.73)	367 (32.71)	0.01		333 (25.81)	346 (36.15)	<0.001
Hyperlipidemia medications	213 (18.93)	237 (21.12)	0.21		268 (20.78)	182 (19.02)	0.33
Hypertension medications	391 (34.76)	445 (39.66)	0.02		435 (33.72)	401 (41.9)	<0.001
Biomarker							
HDL-C (mg/dL)	42.78±11.25	43.41±12.25	0.20		43.99±12.01	41.89±11.32	<0.001
TG (mg/dL)	160.88±152.24	153.10±125.42	0.19		152.25±127.1	163.39±154.53	0.07
LDL-C (mg/dL)	105.55±37.31	102.27±35.19	0.03		105.04±36.09	102.4±36.53	0.09
TC (mg/dL)	181.34±50.8	178.04±46.09	0.11		180.48±48.68	178.64±48.31	0.37
eGFR (ml/min/1.73 m^2^)	78.69±26.26	70.33±26.53	<0.001		77.41±26.02	70.6±27.15	<0.001
FPG (mg/dL)	141.04±50.69	137.46±47.01	0.08		138.27±48.7	140.59±49.19	0.27
HbA1c (%)	7.78±1.71	7.67±1.58	0.11		7.55±1.56	7.95±1.73	<0.001

Differences in continue variables were tested using the Student’s *t* test. Differences in categorical variables were tested using the chi-square test.

SBP: Systolic blood pressure; DBP: Diastolic blood pressure; FPG: Fasting plasma glucose; HbA1c: Hemoglobin A1c; TG: Triglyceride; TC: Total cholesterol; HDL-C: High-density lipoprotein-cholesterol; LDL-C: Low-density lipoprotein-cholesterol; eGFR: Estimated glomerular filtration rate.

“No” = absence and “Yes” = presence of WMH/cerebrovascular abnormality according to the MRI criteria in Methods.

### Associations between glucose variability and brain MRI variables determined using an epidemiologic approach

**Table 2** presents the odds ratios (ORs) and confidence intervals (CIs) for the presence of WMH and cerebrovascular abnormalities associated with various glucose variability measures in the study subjects with type 2 diabetes as observed in an epidemiological study. After adjustment for age and sex or multivariate adjustment, no significant associations were found between any glucose variability measures and the presence of WMH (all p>0.05). With the exception of HbA1c-VIM, all glucose variability measures were significantly associated with the presence of cerebrovascular abnormalities after adjustment for age and sex. Following multivariate adjustment, only AC-CV, AC-SD, AC-VIM, and AC-ARV remained significantly associated with the presence of cerebrovascular abnormalities. The OR per 1 unit change for glucose variability ranged from 1.12 (95% CI: 1.02, 1.23) for AC-ARV to 1.28 (95% CI: 1.15, 1.41) for AC-CV.

**Table 2:** The odds ratios of WMH or cerebrovascular abnormality for various glucose variation measures in patients with type 2 diabetes using observational epidemiologic approach

Glucose variation	WMH OR (95% CI) per standard deviation			Cerebrovascular abnormality OR (95% CI) per standard deviation	
	Ageand sex-adjusted model	Multivariate model		Ageand sex-adjusted model	Multivariate model
AC-CV	1.00 (0.91, 1.09)	0.96 (0.87, 1.06)		1.38 (1.27, 1.50)^***^	1.28 (1.15, 1.41)^***^
AC-SD	0.97 (0.89, 1.06)	0.92 (0.82, 1.02)		1.31 (1.20, 1.43)^***^	1.17 (1.05, 1.31)^**^
AC-VIM	1.03 (0.95, 1.13)	1.01 (0.92, 1.11)		1.32 (1.21, 1.45)^***^	1.27 (1.15, 1.39)^***^
AC-ARV	1.05 (0.96, 1.14)	1.04 (0.95, 1.14)		1.19 (1.09, 1.30)^***^	1.12 (1.02, 1.23)^*^
HbA1c-CV	1.02 (0.92, 1.12)	1.00 (0.89, 1.12)		1.17 (1.06, 1.29)^**^	1.02 (0.91, 1.15)
HbA1c -SD	1.01 (0.91, 1.11)	0.97 (0.85, 1.11)		1.20 (1.08, 1.32)^***^	1.00 (0.88, 1.15)
HbA1c-VIM	1.04 (0.95, 1.15)	1.03 (0.93, 1.14)		1.10 (1.00, 1.21)	1.06 (0.95, 1.17)
HbA1c-ARV	1.00 (0.90, 1.10)	0.96 (0.85, 1.08)		1.18 (1.07, 1.31)^**^	1.02 (0.9, 1.14)

Multivariate model adjusted for age, sex, lifestyle behaviors (smoking, alcohol drinking, physical activity, and BMI), diabetes-related variables (duration of diabetes and type of hypoglycemic drug use), comorbidity (stroke, hypertension, obesity, coronary artery disease, hyperlipidemia, peripheral neuropathy, neuropathy and nephropathy), drug-related variables (cardiovascular medications, hyperlipidemia medications, and hypertension medications) and biomarkers (HDL-C, TG, LDL-C, TC, eGFR, FPG, and HbA1c).

* P<0.05;

** P<0.01;

*** P<0.001; OR: odds ratio; CI: confidence interval.

### Evaluation of MR assumptions 1 and 3 at the SNP level

**Supplementary Table 1** shows the regression coefficients for glucose variability measures and ORs for the presence of WMH and cerebrovascular abnormalities of the significant SNPs meeting SNP-level MR assumptions 1 (all *p* < 0.05) and 3 (all *p* > 0.05) as determined using an additive model. For the SNPs exhibiting negative associations, i.e., those with regression coefficients less than 0 or an ORs less than 1, we reversed the codes to 2, 1, or 0 depending on the number of minor alleles to ensure that the direction of the regression coefficients or ORs consistently remained positive.

MR-Egger regression was performed to test horizontal pleiotropy. The absolute values of the intercepts for the brain MRI variables ranged from 0.0003 to 0.025 (**Supplementary Table 2**). The results of the Cochran’s Q statistics suggest no apparent horizontal pleiotropy because all the intercepts were not significantly different from zero (all *p* > 0.05).

**Supplementary figure 1:**
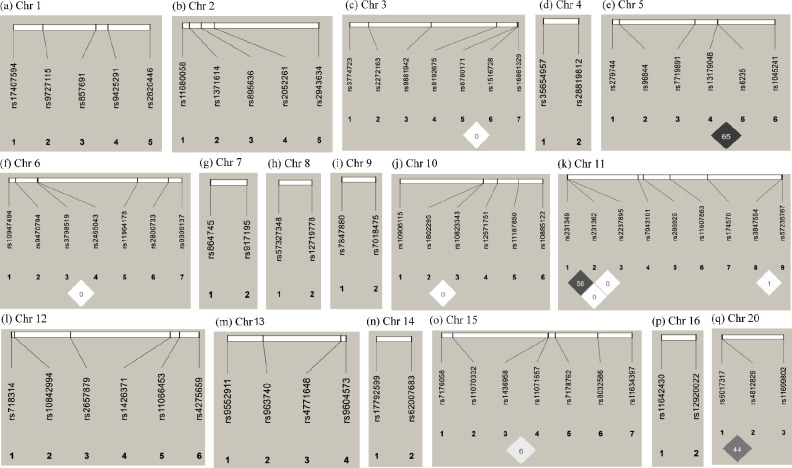
LD analysis of SNPs satisfying MR assumptions 1 and 3

The LD of the SNPs satisfying MR assumptions 1 and 3 was examined. (**Supplementary Figure 1**). The retained SNPs included AC-CV (^19^), AC-SD (^16^), AC-VIM (^19^), and AC-ARV (^20^) for FPG variation measures and HbA1c-CV (^18^), HbA1c-SD (^19^), HbA1c-VIM (^10^), and HbA1c-ARV (^13^) for HbA1c variation measures. We derived weighted and unweighted GRSs using these glucose variability-associated SNPs. The numbers of SNPs at each stage of instrument selection for each GV metric, including the initial SNP set, exclusion based on association with MRI outcomes, weak association with GV, and LD pruning, are listed in **Supplementary Table 3**.

### Evaluation of MR assumptions 1, 2, and 3 at the genetic risk score (GRS) level

We also investigated the GRS-level MR assumption 1, which examined the associations between weighted and unweighted GRSs and glucose variability measures (**Table 3**). Our findings indicate significant positive associations between weighted and unweighted GRSs and glucose variability measures with and without adjustment, thereby meeting assumption 1. As the weighted or unweighted GRS increases, the values of glucose variability measures correspondingly increase.

**Table 3:** Association of genetic risk scores with glucose variation in patients with type 2 diabetes (MR assumption 1)

Glucose variation	Ageand sex-adjusted model				Multivariate model		
	β (SE)	F-value	partial R squared		β (SE)	F-value	partial R squared
**Unweighted**							
GRS_AC-CV_	2.91 (0.30)^***^	91.40^***^	0.039		2.35 (0.27)^***^	74.59^***^	0.033
GRS_AC-SD_	5.95 (0.66)^***^	82.09^***^	0.041		4.06 (0.55)^***^	54.35^***^	0.024
GRS_AC-VIM_	0.01 (0.001)^***^	87.22^***^	0.035		0.01 (0.001)^***^	82.79^***^	0.036
GRS_AC-ARV_	3.56 (0.38)^***^	86.74^***^	0.037		3.09 (0.36)^***^	71.95^***^	0.031
GRS_HbA1c-CV_	1.49 (0.16)^***^	90.08^***^	0.039		1.18 (0.14)^***^	76.48^***^	0.033
GRS_HbA1c -SD_	0.19 (0.02)^***^	90.94^***^	0.037		0.12 (0.02)^***^	55.18^***^	0.024
GRS_HbA1c-VIM_	0.02 (0.003)^***^	56.76^***^	0.038		0.02 (0.003)^***^	87.70^***^	0.038
GRS_HbA1c-ARV_	1.97 (0.24)^***^	66.36^***^	0.032		1.33 (0.22)^***^	72.98^***^	0.032
**Weighted**							
GRS_AC-CV_	2.98 (0.30)^***^	95.57^***^	0.051		2.38 (0.27)^***^	71.35^***^	0.041
GRS_AC-SD_	6.09 (0.66)^***^	85.94^***^	0.054		4.1 (0.55)^***^	56.89^***^	0.033
GRS_AC-VIM_	0.01 (0.001)^***^	91.22^***^	0.051		0.01 (0.001)^***^	52.46^***^	0.031
GRS_AC-ARV_	3.59 (0.38)^***^	88.11^***^	0.054		3.11 (0.36)^***^	37.50^***^	0.022
GRS_HbA1c-CV_	1.54 (0.16)^***^	95.71^***^	0.034		1.20 (0.14)^***^	74.00^***^	0.043
GRS_HbA1c -SD_	0.19 (0.02)^***^	96.21^***^	0.038		0.12 (0.02)^***^	58.85^***^	0.034
GRS_HbA1c-VIM_	0.02 (0.003)^***^	59.66^***^	0.039		0.02 (0.003)^***^	55.48^***^	0.032
GRS_HbA1c-ARV_	2.01 (0.24)^***^	69.26^***^	0.020		1.35 (0.22)^***^	39.00^***^	0.023

Multivariate model adjusted for age, sex, lifestyle behaviors (smoking, alcohol drinking, physical activity, and BMI), diabetes-related variables (duration of diabetes and type of hypoglycemic drug use), comorbidity (stroke, hypertension, obesity, coronary artery disease, hyperlipidemia, peripheral neuropathy, neuropathy and nephropathy), drug-related variables (cardiovascular medications, hyperlipidemia medications, and hypertension medications) and biomarkers (HDL-C, TG, LDL-C, TC, eGFR, FPG, and HbA1c).

* P<0.05;

** P<0.01;

*** P<0.001.

We then explored GRS-level MR assumption 2, which examined the relationship between unweighted and weighted GRSs and covariates. Linear regression analyses were conducted to examine the associations of glucose variability-related unweighted and weighted GRSs with various covariates including lifestyle behaviors, clinical and biochemical markers, sociodemographic factors, and comorbidities. The significant covariates associated with glucose variability measures are presented in **Supplementary Table 4**. These covariates failed to meet assumption 2 and thus were not considered for adjustment in the initial stage of modeling to derive the glucose variability-related scores using GRSs.

**Figure 2:**
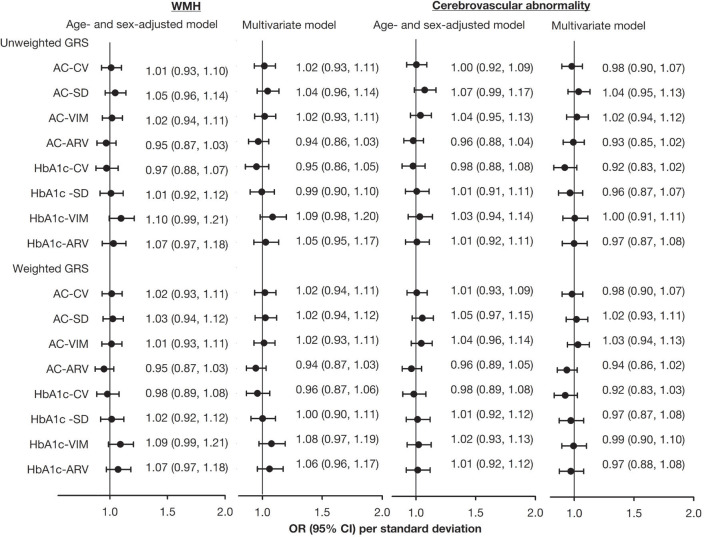
Association of genetic risk scores with WMH or cerebrovascular abnormality in patients with type 2 diabetes for genetic risk score level (MR assumption 3). Multivariate model adjusting for age, sex, lifestyle behaviors, diabetes-related variables, comorbidity, drug-related variables and biomarker.

We also assessed GRS-level MR assumption 3, which investigated the association of unweighted and weighted GRSs with WMH and cerebrovascular abnormalities (**Figure 2**). The unweighted and weighted GRSs for glucose variability measures were not significantly associated with WMH or cerebrovascular abnormalities, regardless of whether age and sex were adjusted or multivariate adjustment was performed, thereby meeting assumption 3.

### Associations between glucose variability and brain MRI variables determined using an MR approach

**Table 4** shows the ORs of the presence of WMH and cerebrovascular abnormalities associated with genetic-related glucose variability, derived from the unweighted and weighted GRSs with and without adjustment. The genetic-related glucose variability scores denoted a genetic predisposition to glucose variability and were obtained by regressing glucose variability measures on the unweighted and weighted GRSs. With respect to the presence of WMH per 1 SD increase in glucose variability scores with residual adjustment, the ORs were significant for AC-CV and AC-VIM in the unweighted GRS and for AC-VIM in the weighted GRS. After the multivariate adjustment, the ORs of the presence of WMH remained significant for AC-VIM in the unweighted GRS. In contrary, the ORs for AC-CV in the unweighted GRS and AC-VIM in the weighted GRS became insignificant after the multivariate adjustment. Among the glucose variability metrics in the unweighted GRS that were significantly associated with multivariate adjustment, only AC-VIM (OR per 1SD: 1.17, 95% CI: 1.08-1.27) was positively associated.

**Table 4:** The odds ratios of WMH or cerebrovascular abnormality for predictive glucose variation derived from unweighted and weighted GRS using MR approach

Glucose variation	WMH OR (95% CI) per standard deviation			Cerebrovascular abnormality OR (95% CI) per standard deviation	
	Model 1	Model 2		Model 1	Model 2
**Unweighted**					
GRS_AC-CV_	1.09 (1.00, 1.18)^*^	1.05 (0.96, 1.14)		1.35 (1.24, 1.47)^***^	1.34 (1.23, 1.47)^***^
GRS_AC-SD_	1.03 (0.95, 1.12)	1.06 (0.97, 1.16)		1.20 (1.10, 1.30)^***^	1.14 (1.04, 1.25)^**^
GRS_AC-VIM_	1.16 (1.07, 1.27)^***^	1.17 (1.08, 1.27)^***^		1.34 (1.23, 1.46)^***^	1.33 (1.22, 1.45)^***^
GRS_AC-ARV_	1.04 (0.96, 1.14)	1.00 (0.91, 1.09)		1.12 (1.03, 1.22)^**^	1.01 (0.92, 1.11)
GRS_HbA1c-CV_	0.96 (0.89, 1.04)	0.96 (0.89, 1.05)		1.36 (1.25, 1.48)^***^	1.36 (1.25, 1.48)^***^
GRS_HbA1c -SD_	1.01 (0.93, 1.09)	1.01 (0.93, 1.10)		1.36 (1.25, 1.48)^***^	1.36 (1.25, 1.48)^***^
GRS_HbA1c-VIM_	1.02 (0.94, 1.11)	1.03 (0.95, 1.12)		1.37 (1.26, 1.49)^***^	1.37 (1.26, 1.50)^***^
GRS_HbA1c-ARV_	0.98 (0.91, 1.07)	0.98 (0.91, 1.07)		1.34 (1.23, 1.46)^***^	1.34 (1.23, 1.46)^***^
**Weighted**					
GRS_AC-CV_	1.08 (0.99, 1.17)	1.05 (0.96, 1.15)		1.21 (1.11, 1.31)^***^	1.14 (1.04, 1.25)^**^
GRS_AC-SD_	1.03 (0.95, 1.12)	1.06 (0.97, 1.16)		1.21 (1.11, 1.32)^***^	1.16 (1.06, 1.27)^**^
GRS_AC-VIM_	1.10 (1.02, 1.20)^*^	1.03 (0.94, 1.12)		1.28 (1.18, 1.40)^***^	1.23 (1.13, 1.35)^***^
GRS_AC-ARV_	1.05 (0.97, 1.14)	1.02 (0.93, 1.11)		1.23 (1.13, 1.33)^***^	1.16 (1.06, 1.27)^**^
GRS_HbA1c-CV_	0.96 (0.89, 1.05)	0.96 (0.89, 1.05)		1.36 (1.25, 1.48)^***^	1.37 (1.25, 1.49)^***^
GRS_HbA1c -SD_	0.95 (0.87, 1.03)	0.95 (0.87, 1.04)		1.20 (1.10, 1.30)^***^	1.12 (1.02, 1.23)^*^
GRS_HbA1c-VIM_	1.02 (0.94, 1.11)	1.03 (0.95, 1.12)		1.37 (1.25, 1.49)^***^	1.37 (1.26, 1.5)^***^
GRS_HbA1c-ARV_	0.94 (0.86, 1.02)	0.95 (0.87, 1.04)		1.18 (1.08, 1.28)^***^	1.11 (1.02, 1.22)^*^

Model 1 adjusted for residuals.

Model 2 adjusting for residual, PCA and confounding variables.

* P<0.05;

** P<0.01;

*** P<0.001; OR: odds ratio; CI: confidence interval.

The ORs of the presence of cerebrovascular abnormalities per 1 SD increase in glucose variability scores with residual adjustment were significant for all the unweighted and weighted GRSs. After multivariate adjustment, all the significant ORs of the presence of cerebrovascular abnormalities remained significant for the unweighted and weighted GRSs, except for the AC-ARV unweighted GRS. Among the glucose variability metrics in the unweighted GRS that were significantly associated with multivariate adjustment, the OR per 1 unit change for glucose variability measures ranged from 1.14 (1.04, 1.25) for AC-SD to 1.37 (1.26, 1.50) for HbA1c-VIM.

## DISCUSSION

In this study, the associations between glucose variability and brain MRI indicators were thoroughly assessed using MR and epidemiologic approaches. When applied the epidemiologic approach, we did not observe any significant associations between glucose variability and WMH. However, we found significant associations between all the FPG variability metrics and cerebrovascular abnormalities. With the MR approach, the absence of significant associations between the weighted or unweighted genetic risk scores and WMH or cerebrovascular abnormalities supports the validity of MR assumption 3, suggesting no evidence of direct genetic effects on the outcomes. In contrast, the two-stage MR analyses revealed a significant association between genetically predicted FPG variability, as measured by AC-VIM, and WMH, with a 17% increase in odds per 1 SD increase when the unweighted GRS was used. These findings reflect an indirect effect of genetic instruments on WMH mediated through glycemic variability rather than a direct instrument-outcome association. The association was not significant for AC-VIM in the weighted model after the multivariate adjustment, indicating the potential specificity of this metric in the unweighted genetic context. For cerebrovascular abnormalities, the glucose variability metrics in the unweighted and weighted GRS models showed consistent and significant associations after full adjustment, with the exception of the AC-ARV in the unweighted GRS group. Among the significant metrics in the unweighted GRS group, the strongest association was observed with HbA1c-VIM (OR per 1 unit increase: 1.37; 95% CI: 1.26-1.50), followed by AC-SD (OR: 1.14; 95% CI: 1.04-1.25), highlighting the clinical relevance of glycemic variability as an independent risk factor for cerebrovascular pathology.

These findings underscore the importance of glycemic variability - beyond average glucose levels-as a potential independent risk factor for cerebral small vessel disease and cerebrovascular abnormalities. The observed association between FPG variability (AC-VIM) and WMH, particularly in the context of unweighted genetic risk, suggests that fluctuations in fasting glucose levels may contribute to subclinical brain injury. Moreover, the consistent and significant associations between cerebrovascular abnormalities and multiple glucose variability metrics, especially the strong link with HbA1c-VIM, support the hypothesis that long-term glycemic instability may play a causal role in vascular brain damage. From a clinical perspective, these results highlight the need to monitor and manage chronic hyperglycemia and glycemic fluctuations, particularly in individuals at high genetic risk, as part of strategies to reduce the burden of cerebrovascular disease and cognitive decline.

Only a few epidemiological and MR studies have investigated the association between visit-to-visit glucose variability and brain MRI parameters (^14^-^16^). One epidemiological study examined individuals with type 1 diabetes (^14^), and another focused on APOE4 genotype carriers with type 2 diabetes (^15^). One MR study assessed the relationship between HbA1c levels - rather than HbA1c variability-and WMH (^16^). In a study of individuals with type 1 diabetes, no association between HbA1c variability and cerebral small vessel disease was found among 189 neurologically asymptomatic participants (^14^). In contrast, our work demonstrated a consistent association between glucose variability and cerebrovascular abnormalities. This discrepancy may be attributed to our large sample size and the use of a composite outcome measure that includes stenosis or occlusion of major intracranial or internal carotid arteries, aneurysms, lacunar or small infarctions, intracerebral hemorrhage, and lobar infarctions-features that may increase the prevalence and statistical power of a study. In the investigation of WMH, previous epidemiological and MR studies reported differing findings: the former identified associations (^15^), and the latter did not (^16^). In an investigation of individuals aged 65 years and older with type 2 diabetes and carrying the APOE4 genotype (n = 124), HbA1c variability was found to be significantly associated with high WMH burden in APOE4 carriers (^15^). Two-sample MR analysis revealed no significant association between genetic liability and type 2 diabetes or between HbA1c levels and WMH (^16^). Although a significant association between FPG-VIM and WMH was found in this work, most other glucose variability metrics were consistent with the MR findings. Substantial methodological differences were noted between the above studies and the current study. These epidemiological studies focused on older adults with type 2 diabetes who carry the APOE4 allele; despite having a relatively large sample size, our work focused on asymptomatic adults over the age of 30 years with type 2 diabetes. Thus, the prevalence of WMH in our study was relatively low, which may have resulted in limited statistical power. Further research is warranted to clarify these associations.

Several limitations of this study should be acknowledged. First, the glucose-related variables - FPG and HbA1c - were derived from routine clinical monitoring, resulting in variability in the number of measurements obtained across the participants. To mitigate this issue, we adjusted for the number of FPG and HbA1c measurements when calculating variability indices. Moreover, these glucose measurements were collected prior to the brain MRI assessments, and the duration between the two procedures may not have been sufficient to capture the full temporal relationship between glucose variability and brain MRI outcomes. Second, the study sample may not be representative of the broad type 2 diabetes population; the study subjects may have undergone brain MRI for specific clinical indications. In clinical settings, brain MRI is often performed in the presence of suspected or known cerebrovascular pathology. Hence, the participants included in this analysis may have had underlying cerebrovascular symptoms or risk factors, limiting the generalizability of the findings to individuals with type 2 diabetes who do not present with such concerns. Third, cerebrovascular abnormalities were analyzed as a composite binary variable encompassing heterogeneous lesion types, including large-vessel stenosis or occlusion, aneurysms, lacunar infarctions, intracerebral hemorrhage, and lobar infarctions. Owing to the structure of the Clinical Research Data Repository and the pre-specified data request at the time of application, lesion-specific information was not available for further stratified or severity-based analyses. The use of a composite endpoint may introduce heterogeneity, as these lesions differ in terms of their underlying pathophysiology and clinical significance. Such heterogeneity is more likely to bias associations toward the null, potentially diluting lesion-specific effects of glycemic variability rather than inflating them. Therefore, the observed associations should be interpreted as conservative estimates of the relationship between glycemic variability and overall cerebrovascular disease burden. Future studies with access to detailed neuroimaging phenotypes are warranted to disentangle lesion-specific associations and to evaluate whether glycemic variability differentially influences ischemic, hemorrhagic, and large-vessel cerebrovascular processes. Fourth, although WMH is a canonical marker of cerebral small-vessel disease, WMH was available only as a binary measure in the current dataset. Information on WMH volume or severity grade was not accessible because of the structure of the clinical imaging data repository. As a result, we were unable to examine dose-response relationships or formally assess effect modification by WMH burden in the association between glycemic variability and other cerebrovascular abnormalities. Future studies incorporating quantitative or graded WMH measures may provide further insights into whether the impact of glycemic variability differs across levels of small-vessel disease burden. Fifth, markers of neurodegenerative pathology, such as amyloid biomarkers or cognitive performance measures, were not available in this cohort. Although the APOE genotype was included in the genetic analyses, the lack of phenotypic neurodegenerative data limits our ability to distinguish between purely vascular pathology and mixed vascular-neurodegenerative processes underlying the observed MRI findings. Finally, causal inferences cannot be drawn because this cohort study was observational. The findings represent associations rather than definitive causal relationships. In addition, the association between glucose variability and WMH was not consistently observed across the epidemiological and MR approaches. For the MR analysis, this inconsistency may be attributed to the use of weak instrumental variables, which could limit the strength and reliability of the causal estimates.

In conclusion, this study thoroughly evaluated the associations between glucose variability and brain MRI outcomes using epidemiological and MR approaches. Epidemiological and MR analyses revealed no significant association between glucose variability and WMH. Meanwhile, FPG and HbA1c variability were significantly associated with cerebrovascular abnormalities across most metrics. These findings emphasize the importance of monitoring and managing glucose variability, beyond measuring average glucose levels, in persons with type 2 diabetes to potentially mitigate cerebrovascular risk.

## Figures and Tables

**Supplementary table 1:** SNP-level characteristics and associations for GV metrics

SNP ID	Chromosome	Position	Effect allele	Other allele	EAF	Effect size on GV (β (SE)	P-value	OR with WMH	OR with cerebrovascular Abnormality
**GRSAC-CV (19 SNPs)**									
rs857691	1	158626378	C	T	0.55	-0.88 (0.45)	0.049	0.99 (0.88, 1.11)	0.99 (0.88, 1.12)
rs1371614	2	27152874	C	T	0.83	-1.29 (0.58)	0.027	1.12 (0.96, 1.31)	0.93 (0.79, 1.09)
rs9881942	3	123082416	G	A	0.71	-1.29 (0.49)	0.008	1.02 (0.90, 1.16)	1.01 (0.88, 1.15)
rs28819812	4	157652753	A	C	0.25	1.15 (0.50)	0.023	0.91 (0.79, 1.04)	0.96 (0.84, 1.10)
rs96844	5	56196604	G	A	0.81	-1.15 (0.56)	0.040	0.94 (0.81, 1.09)	1.04 (0.90, 1.21)
rs13179048	5	95542726	C	A	0.65	-0.92 (0.46)	0.047	1.04 (0.92, 1.18)	1.01 (0.89, 1.15)
rs10947494	6	34263743	G	A	0.27	1.10 (0.49)	0.026	1.07 (0.94, 1.22)	0.94 (0.83, 1.08)
rs2800733	6	127416930	A	G	0.89	-1.48 (0.71)	0.037	1.20 (1.00, 1.45)	0.92 (0.76, 1.12)
rs7943101	11	32460873	T	C	0.07	1.84 (0.87)	0.034	1.16 (0.92, 1.47)	0.94 (0.74, 1.18)
rs286925	11	34642668	G	A	0.40	1.20 (0.45)	0.008	0.92 (0.81, 1.03)	0.91 (0.81, 1.03)
rs3847554	11	92668826	C	T	0.44	0.87 (0.43)	0.047	1.02 (0.90, 1.14)	0.91 (0.81, 1.02)
rs4275659	12	123447928	C	T	0.72	-0.98 (0.49)	0.045	0.89 (0.78, 1.02)	0.99 (0.87, 1.13)
rs963740	13	51096095	A	T	0.35	1.20 (0.46)	0.010	0.98 (0.87, 1.11)	1.04 (0.92, 1.18)
rs17792599	14	21770681	G	A	0.10	1.56 (0.76)	0.039	1.09 (0.89, 1.33)	1.01 (0.83, 1.24)
rs7176058	15	39464167	A	G	0.52	-0.97 (0.43)	0.025	1.1 (0.98, 1.24)	0.99 (0.89, 1.12)
rs11642430	16	30045789	C	G	0.56	-0.93 (0.43)	0.032	0.94 (0.84, 1.05)	0.90 (0.8,0 1.01)
rs12920022	16	89564055	T	A	0.87	-1.39 (0.65)	0.033	0.93 (0.78, 1.11)	1.06 (0.89, 1.27)
rs6017317	20	42946966	T	G	0.56	-1.03 (0.44)	0.020	0.91 (0.81, 1.02)	0.89 (0.79, 1.00)
rs112915006	22	50604696	G	A	0.10	1.88 (0.71)	0.008	1.17 (0.97, 1.41)	1.11 (0.92, 1.34)
**GRSAC-SD (16 SNPs)**									
rs11680058	2	16574669	A	G	0.88	-3.10 (1.47)	0.035	0.96 (0.80, 1.15)	1.09 (0.91, 1.31)
rs9881942	3	123082416	G	A	0.71	-3.10 (1.05)	0.003	1.02 (0.90, 1.16)	1.01 (0.88, 1.15)
rs28819812	4	157652753	A	C	0.25	2.21 (1.09)	0.043	0.91 (0.79, 1.04)	0.96 (0.84, 1.10)
rs96844	5	56196604	G	A	0.81	-3.04 (1.20)	0.012	0.94 (0.81, 1.09)	1.04 (0.90, 1.21)
rs7719891	5	86577352	A	G	0.51	-2.35 (0.93)	0.012	1.02 (0.91, 1.15)	1.00 (0.89, 1.13)
rs9399137	6	135419018	C	T	0.23	2.37 (1.16)	0.042	1.09 (0.94, 1.26)	0.97 (0.84, 1.12)
rs7847880	9	20662703	C	T	0.76	-2.93 (1.11)	0.008	0.98 (0.85, 1.12)	0.93 (0.81, 1.07)
rs286925	11	34642668	G	A	0.40	2.34 (0.97)	0.015	0.92 (0.81, 1.03)	0.91 (0.81, 1.03)
rs11607883	11	45839709	A	G	0.24	2.49 (1.12)	0.026	0.97 (0.85, 1.12)	0.99 (0.86, 1.14)
rs963740	13	51096095	A	T	0.35	2.98 (1.00)	0.003	0.98 (0.87, 1.11)	1.04 (0.92, 1.18)
rs7176058	15	39464167	A	G	0.52	-2.25 (0.93)	0.016	1.10 (0.98, 1.24)	0.99 (0.89, 1.12)
rs11070332	15	41809205	G	A	0.84	-2.72 (1.31)	0.038	0.93 (0.79, 1.10)	0.90 (0.76, 1.06)
rs11634397	15	80432222	G	A	0.08	3.58 (1.72)	0.038	0.89 (0.72, 1.10)	1.09 (0.88, 1.35)
rs6017317	20	42946966	T	G	0.56	-3.00 (0.95)	0.002	0.91 (0.81, 1.02)	0.89 (0.79, 1.00)
rs4812829	20	42989267	G	A	0.55	-1.91 (0.96)	0.047	0.94 (0.84, 1.06)	0.91 (0.80, 1.02)
rs112915006	22	50604696	G	A	0.10	3.56 (1.52)	0.019	1.17 (0.97, 1.41)	1.11 (0.92, 1.34)
**GRSAC-VIM (19 SNPs)**									
rs857691	1	158626378	C	T	0.55	-0.004 (0.002)	0.026	0.99 (0.88, 1.11)	0.99 (0.88, 1.12)
rs2820446	1	219748818	G	C	0.23	0.004 (0.002)	0.049	0.96 (0.84, 1.10)	1.07 (0.93, 1.22)
rs2943634	2	227068080	A	C	0.08	0.007 (0.003)	0.016	0.88 (0.71, 1.10)	1.09 (0.87, 1.35)
rs13179048	5	95542726	C	A	0.65	-0.005 (0.002)	0.009	1.04 (0.92, 1.18)	1.01 (0.89, 1.15)
rs6235	5	95728898	C	G	0.67	-0.004 (0.002)	0.034	1.05 (0.93, 1.19)	1.00 (0.88, 1.14)
rs10947494	6	34263743	G	A	0.27	0.004 (0.002)	0.021	1.07 (0.94, 1.22)	0.94 (0.83, 1.08)
rs57327348	8	10808687	T	A	0.09	0.007 (0.003)	0.024	0.93 (0.75, 1.15)	1.01 (0.82, 1.25)
rs11187850	10	96068480	G	A	0.20	0.004 (0.002)	0.037	1.02 (0.88, 1.18)	1.12 (0.97, 1.30)
rs718314	12	26453283	G	A	0.72	-0.004 (0.002)	0.032	0.99 (0.87, 1.13)	0.99 (0.87, 1.13)
rs1426371	12	108629780	A	G	0.57	-0.004 (0.002)	0.022	1.03 (0.91, 1.16)	1.06 (0.94, 1.20)
rs4275659	12	123447928	C	T	0.72	-0.004 (0.002)	0.018	0.89 (0.78, 1.02)	0.99 (0.87, 1.13)
rs4771648	13	110431626	A	G	0.45	0.004 (0.002)	0.008	1.07 (0.95, 1.20)	1.02 (0.91, 1.15)
rs9604573	13	114542858	A	G	0.22	0.004 (0.002)	0.047	1.01 (0.88, 1.17)	1.02 (0.88, 1.18)
rs17792599	14	21770681	G	A	0.10	0.006 (0.003)	0.035	1.09 (0.89, 1.33)	1.01 (0.83, 1.24)
rs62007683	14	103894071	T	G	0.12	0.006 (0.003)	0.027	1.16 (0.97, 1.38)	1.05 (0.88, 1.26)
rs11642430	16	30045789	C	G	0.56	-0.005 (0.002)	0.004	0.94 (0.84, 1.05)	0.90 (0.80, 1.01)
rs34855406	17	40731411	C	G	0.24	0.005 (0.002)	0.013	0.91 (0.79, 1.04)	1.02 (0.89, 1.17)
rs11672660	19	46180184	T	C	0.20	0.004 (0.002)	0.043	1.07 (0.93, 1.24)	0.99 (0.86, 1.15)
rs112915006	22	50604696	G	A	0.10	0.006 (0.003)	0.037	1.17 (0.97, 1.41)	1.11 (0.92, 1.34)
**GRSAC-ARV (20 SNPs)**									
rs9425291	1	172312769	G	A	0.87	-1.63 (0.82)	0.046	0.99 (0.83, 1.17)	1.11 (0.93, 1.32)
rs2820446	1	219748818	G	C	0.23	1.32 (0.65)	0.042	0.96 (0.84, 1.10)	1.07 (0.93, 1.22)
rs1371614	2	27152874	C	T	0.83	-1.93 (0.73)	0.008	1.12 (0.96, 1.31)	0.93 (0.79, 1.09)
rs16861329	3	186666461	T	C	0.19	1.52 (0.72)	0.034	0.95 (0.82, 1.11)	0.97 (0.83, 1.13)
rs28819812	4	157652753	A	C	0.25	1.28 (0.63)	0.044	0.91 (0.79, 1.04)	0.96 (0.84, 1.10)
rs96844	5	56196604	G	A	0.81	-1.66 (0.70)	0.018	0.94 (0.81, 1.09)	1.04 (0.90, 1.21)
rs11964178	6	109562035	A	G	0.78	-1.48 (0.65)	0.024	1.13 (0.99, 1.30)	0.97 (0.84, 1.12)
rs7847880	9	20662703	C	T	0.76	-1.34 (0.65)	0.038	0.98 (0.85, 1.12)	0.93 (0.81, 1.07)
rs7018475	9	22137685	G	T	0.35	1.20 (0.58)	0.037	0.95 (0.85, 1.08)	0.92 (0.81, 1.04)
rs1802295	10	70931474	T	C	0.11	2.35 (0.88)	0.008	1.02 (0.84, 1.23)	1.10 (0.91, 1.32)
rs12571751	10	80942631	A	G	0.61	-1.12 (0.56)	0.045	0.97 (0.86, 1.09)	1.00 (0.88, 1.12)
rs231349	11	2672821	C	T	0.93	-2.19 (1.08)	0.042	1.06 (0.84, 1.33)	1.05 (0.83, 1.32)
rs231362	11	2691471	G	A	0.91	-2.45 (0.98)	0.013	0.98 (0.80, 1.21)	1.12 (0.91, 1.38)
rs3847554	11	92668826	C	T	0.44	1.29 (0.55)	0.018	1.02 (0.90, 1.14)	0.91 (0.81, 1.02)
rs1426371	12	108629780	A	G	0.57	-1.34 (0.56)	0.017	1.03 (0.91, 1.16)	1.06 (0.94, 1.20)
rs4275659	12	123447928	C	T	0.72	-1.31 (0.62)	0.034	0.89 (0.78, 1.02)	0.99 (0.87, 1.13)
rs7176058	15	39464167	A	G	0.52	-1.33 (0.54)	0.015	1.10 (0.98, 1.24)	0.99 (0.89, 1.12)
rs1436958	15	62338797	G	T	0.65	-1.15 (0.57)	0.045	1.03 (0.91, 1.16)	0.99 (0.88, 1.12)
rs11071657	15	62433962	A	G	0.65	-1.16 (0.58)	0.045	1.10 (0.97, 1.24)	1.12 (0.99, 1.27)
rs8032586	15	73081067	T	C	0.51	-1.15 (0.55)	0.035	0.93 (0.83, 1.05)	0.96 (0.86, 1.08)
**GRSHbA1c-CV (18 SNPs)**									
rs17407594	1	66170362	A	G	0.09	0.88 (0.41)	0.032	0.92 (0.72, 1.18)	0.95 (0.75, 1.22)
rs2052261	2	65355270	A	G	0.67	-0.50 (0.24)	0.039	1.07 (0.93, 1.23)	1.06 (0.92, 1.23)
rs3774723	3	63962339	G	A	0.61	-0.50 (0.23)	0.034	1.00 (0.87, 1.15)	1.10 (0.96, 1.27)
rs2272163	3	77671721	A	C	0.42	0.58 (0.23)	0.014	1.00 (0.87, 1.15)	0.97 (0.84, 1.12)
rs8192675	3	170724883	C	T	0.23	0.57 (0.27)	0.034	0.96 (0.82, 1.13)	0.93 (0.79, 1.10)
rs1516728	3	185829891	A	T	0.40	0.47 (0.24)	0.046	0.95 (0.82, 1.09)	1.00 (0.86, 1.15)
rs279744	5	53412620	A	C	0.26	0.59 (0.26)	0.023	1.03 (0.88, 1.20)	1.11 (0.95, 1.30)
rs1045241	5	118729286	T	C	0.15	0.63 (0.32)	0.048	0.94 (0.78, 1.13)	0.89 (0.73, 1.08)
rs864745	7	28180556	T	C	0.80	-0.63 (0.29)	0.031	1.10 (0.93, 1.31)	0.96 (0.81, 1.15)
rs12719778	8	145879883	C	T	0.35	0.73 (0.24)	0.003	1.03 (0.89, 1.19)	0.98 (0.85, 1.14)
rs10906115	10	12314997	A	G	0.62	-0.52 (0.23)	0.025	0.95 (0.83, 1.09)	1.03 (0.89, 1.18)
rs10885122	10	113042093	G	T	0.94	-1.12 (0.48)	0.019	1.18 (0.89, 1.56)	0.94 (0.70, 1.25)
rs10842994	12	27965150	T	C	0.19	0.57 (0.28)	0.043	1.05 (0.89, 1.24)	1.01 (0.85, 1.19)
rs2657879	12	56865338	G	A	0.09	0.96 (0.39)	0.016	1.11 (0.88, 1.40)	1.16 (0.92, 1.47)
rs9552911	13	23864657	A	G	0.25	0.68 (0.27)	0.010	1.09 (0.93, 1.28)	0.94 (0.80, 1.11)
rs7178762	15	63871292	C	T	0.92	-1.07 (0.43)	0.014	1.04 (0.80, 1.34)	1.13 (0.87, 1.47)
rs11642430	16	30045789	C	G	0.56	-0.50 (0.23)	0.030	0.97 (0.84, 1.10)	0.92 (0.80, 1.05)
rs11699802	20	48832135	T	C	0.54	-0.55 (0.23)	0.018	1.07 (0.94, 1.23)	0.96 (0.84, 1.10)
**GRSHbA1c-SD (19 SNPs)**									
rs2272163	3	77671721	A	C	0.42	0.06 (0.03)	0.036	1.00 (0.87, 1.15)	0.97 (0.84, 1.12)
rs6780171	3	185503456	A	T	0.26	0.07 (0.03)	0.039	0.97 (0.83, 1.13)	0.97 (0.83, 1.14)
rs35654957	4	1010077	C	T	0.27	0.07 (0.03)	0.023	1.08 (0.92, 1.25)	1.14 (0.98, 1.34)
rs1045241	5	118729286	T	C	0.15	0.08 (0.04)	0.042	0.94 (0.78, 1.13)	0.89 (0.73, 1.08)
rs9470794	6	38106844	C	T	0.35	0.06 (0.03)	0.031	0.93 (0.81, 1.07)	1.03 (0.89, 1.19)
rs2465043	6	51180765	G	A	0.39	0.06 (0.03)	0.026	1.02 (0.89, 1.17)	0.90 (0.79, 1.04)
rs864745	7	28180556	T	C	0.80	-0.08 (0.04)	0.028	1.10 (0.93, 1.31)	0.96 (0.81, 1.15)
rs12719778	8	145879883	C	T	0.35	0.10 (0.03)	0.002	1.03 (0.89, 1.19)	0.98 (0.85, 1.14)
rs10906115	10	12314997	A	G	0.62	-0.06 (0.03)	0.046	0.95 (0.83, 1.09)	1.03 (0.89, 1.18)
rs10823343	10	71091013	A	G	0.88	-0.09 (0.04)	0.036	0.88 (0.72, 1.09)	1.02 (0.82, 1.26)
rs10885122	10	113042093	G	T	0.94	-0.14 (0.06)	0.022	1.18 (0.89, 1.56)	0.94 (0.70, 1.25)
rs2237895	11	2857194	A	C	0.61	-0.06 (0.03)	0.045	0.95 (0.83, 1.10)	1.01 (0.88, 1.17)
rs174570	11	61597212	T	C	0.59	-0.06 (0.03)	0.038	1.09 (0.95, 1.25)	0.98 (0.85, 1.12)
rs10842994	12	27965150	T	C	0.19	0.07 (0.04)	0.049	1.05 (0.89, 1.24)	1.01 (0.85, 1.19)
rs2657879	12	56865338	G	A	0.09	0.14 (0.05)	0.005	1.11 (0.88, 1.40)	1.16 (0.92, 1.47)
rs9552911	13	23864657	A	G	0.25	0.07 (0.03)	0.028	1.09 (0.93, 1.28)	0.94 (0.80, 1.11)
rs7178762	15	63871292	C	T	0.92	-0.12 (0.05)	0.022	1.04 (0.80, 1.34)	1.13 (0.87, 1.47)
rs11642430	16	30045789	C	G	0.56	-0.06 (0.03)	0.031	0.97 (0.84, 1.10)	0.92 (0.80, 1.05)
rs11699802	20	48832135	T	C	0.54	-0.07 (0.03)	0.024	1.07 (0.94, 1.23)	0.96 (0.84, 1.10)
GRSHbA1c -VIM (10 SNPs)									
rs17407594	1	66170362	A	G	0.09	0.02 (0.007)	0.004	0.92 (0.72, 1.18)	0.95 (0.75, 1.22)
rs895636	2	45188353	C	T	0.58	-0.01 (0.004)	0.043	0.98 (0.85, 1.12)	0.94 (0.82, 1.08)
rs2272163	3	77671721	A	C	0.42	0.01 (0.004)	0.008	1.00 (0.87, 1.15)	0.97 (0.84, 1.12)
rs3798519	6	50788778	A	C	0.78	-0.01 (0.004)	0.035	0.90 (0.77, 1.06)	0.88 (0.75, 1.04)
rs917195	7	30728452	C	T	0.84	-0.01 (0.005)	0.014	0.92 (0.77, 1.11)	1.05 (0.87, 1.27)
rs12719778	8	145879883	C	T	0.35	0.01 (0.004)	0.009	1.03 (0.89, 1.19)	0.98 (0.85, 1.14)
rs10906115	10	12314997	A	G	0.62	-0.01 (0.004)	0.038	0.95 (0.83, 1.09)	1.03 (0.89, 1.18)
rs57235767	11	93013531	T	C	0.17	0.01 (0.005)	0.013	1.08 (0.91, 1.29)	1.08 (0.90, 1.29)
rs9552911	13	23864657	A	G	0.25	0.01 (0.004)	0.013	1.09 (0.93, 1.28)	0.94 (0.80, 1.11)
rs11642430	16	30045789	C	G	0.56	-0.01 (0.004)	0.023	0.97 (0.84, 1.10)	0.92 (0.80, 1.05)
GRSHbA1c-ARV (13 SNPs)									
rs9727115	1	99177253	A	G	0.36	0.88 (0.36)	0.015	1.04 (0.90, 1.19)	1.00 (0.87, 1.16)
rs6780171	3	185503456	A	T	0.26	0.93 (0.40)	0.020	0.97 (0.83, 1.13)	0.97 (0.83, 1.14)
rs2465043	6	51180765	G	A	0.39	0.78 (0.35)	0.027	1.02 (0.89, 1.17)	0.90 (0.79, 1.04)
rs864745	7	28180556	T	C	0.80	-0.93 (0.44)	0.037	1.10 (0.93, 1.31)	0.96 (0.81, 1.15)
rs12719778	8	145879883	C	T	0.35	1.04 (0.37)	0.005	1.03 (0.89, 1.19)	0.98 (0.85, 1.14)
rs7847880	9	20662703	C	T	0.76	-0.82 (0.41)	0.046	1.01 (0.86, 1.18)	0.88 (0.75, 1.03)
rs10823343	10	71091013	A	G	0.88	-1.19 (0.55)	0.029	0.88 (0.72, 1.09)	1.02 (0.82, 1.26)
rs2237895	11	2857194	A	C	0.61	-0.94 (0.36)	0.009	0.95 (0.83, 1.10)	1.01 (0.88, 1.17)
rs2657879	12	56865338	G	A	0.09	1.67 (0.60)	0.006	1.11 (0.88, 1.40)	1.16 (0.92, 1.47)
rs11066453	12	113365621	G	A	0.18	0.96 (0.45)	0.034	0.92 (0.77, 1.09)	0.96 (0.80, 1.15)
rs9552911	13	23864657	A	G	0.25	0.86 (0.41)	0.034	1.09 (0.93, 1.28)	0.94 (0.80, 1.11)
rs7178762	15	63871292	C	T	0.92	-1.40 (0.66)	0.035	1.04 (0.80, 1.34)	1.13 (0.87, 1.47)
rs11642430	16	30045789	C	G	0.56	-0.81 (0.35)	0.021	0.97 (0.84, 1.10)	0.92 (0.80, 1.05)

**Supplementary table 2:** The Intercepts for the associations between glucose variation and WMH or cerebrovascular abnormality from MR-Egger regression

Glucose variation	MR-Egger regression				
	WMH			cerebrovascular abnormality	
	Estimate	p		Estimate	p
AC-CV	0.017	0.45		-0.025	0.10
AC-SD	-0.019	0.36		-0.011	0.55
AC-VIM	0.006	0.78		0.011	0.35
AC-ARV	-0.009	0.60		0.0003	0.98
HbA1c-CV	0.025	0.10		-0.001	0.95
HbA1c -SD	0.011	0.52		-0.009	0.64
HbA1c-VIM	-0.018	0.38		-0.018	0.41
HbA1c-ARV	-0.014	0.46		-0.015	0.49
	IVW heterogeneity test				
AC-CV	Cochran’s Q test	p value		Cochran’s Q test	p value
AC-SD	18.72	0.41		11.91	0.85
AC-VIM	13.15	0.59		10.60	0.78
AC-ARV	13.32	0.77		6.63	0.99
HbA1c-CV	12.94	0.84		11.77	0.90
HbA1c -SD	9.24	0.93		10.98	0.86
HbA1c-VIM	11.57	0.87		13.44	0.76
HbA1c-ARV	4.71	0.86		5.40	0.80

**Supplementary table 3:** The number of SNPs at each stage of instrument selection for each GV metric, including the initial SNP set, exclusion based on association with MRI outcomes, weak association with GV, and LD pruning

Item	Glucose variation							
	AC-CV	AC-SD	AC-VIM	AC-ARV	HbA1c-CV	HbA1c -SD	HbA1c-VIM	HbA1c-ARV
Initial SNP set	536	536	536	536	536	536	536	536
Associations with MRI outcomes (MR assumption 3)	502	502	502	502	502	502	502	502
Exclusions based on weak association with GV (MR assumption 1)	21	17	27	25	20	26	11	18
LD pruning	19	16	19	20	18	19	10	13

**Supplementary table 4:** List of confounding variables associated with genetic risk scores in patients with type 2 diabetes

Glucose variation	Confounding variables (p<0.05)
GRS_AC-CV_	Duration of diabetes
GRS_AC-SD_	HbA1c, stroke
GRS_AC-VIM_	Coronary artery disease
GRS_AC-ARV_	eGFR, AC, HbA1c
GRS_HbA1c-CV_	Hypertension
GRS_HbA1c-SD_	Coronary artery disease
GRS_HbA1c-VIM_	BMI, AC
GRS_HbA1c-ARV_	
wGRS_AC-CV_	Duration of diabetes, HbA1c, obesity
wGRS_AC-SD_	HbA1c
wGRS_AC-VIM_	Age, eGFR
wGRS_AC-ARV_	TG, eGFR, AC
wGRS_HbA1c-CV_	BMI, hypertension
wGRS_HbA1c-SD_	Duration of diabetes, coronary artery disease, hyperlipidemia medications, HbA1c
wGRS_HbA1c-VIM_	BMI, hypertension, AC
wGRS_HbA1c-ARV_	Coronary artery disease, HbA1c

## Data Availability

datasets related to this article will be available upon request to the corresponding author.
